# Circulating tumor DNA in colorectal cancer: biology, methods and applications

**DOI:** 10.1007/s12672-025-02220-z

**Published:** 2025-04-01

**Authors:** Han Chen, Yang An, Chentong Wang, Jiaolin Zhou

**Affiliations:** Department of General Surgery, Peking Union Medical College Hospital, Chinese Academy of Medical Sciences and Peking Union Medical College, No.1, Shuaifuyuan, Beijing, 100730 China

**Keywords:** Colorectal cancer, Liquid biopsy, Circulating tumor DNA, Response, Prognosis

## Abstract

In the practice of colorectal cancer (CRC), traditional tumor tissue analysis is limited by intratumoral and intertumoral heterogeneity and its invasive nature. Circulating tumor DNA (ctDNA) analysis, a promising liquid biopsy approach, has been increasingly explored in clinical studies. Biologically, ctDNA is characterized by tumor-specific diversity and rapid clearance from circulation, enabling real-time, dynamic, and repeatable assessments. Technologically, PCR- and NGS-based downstream analysis methods have been developed and validated. However, variables in pre-analytical and analytical procedures underscores the need for standardized protocols. Compared with clinicopathology-based risk stratification, ctDNA-based molecular residual disease detection has demonstrated significant potential in guiding treatment decisions. Qualitative and quantitative changes in ctDNA have also shown predictive and prognostic value during neoadjuvant or adjuvant treatment, as well as in later-line treatment for metastatic CRC. Specific molecular aberrations in ctDNA can not only assist in identifying candidates for targeted therapies but also reveal resistance mechanisms. Additionally, emerging research is exploring the potential of ctDNA in early cancer detection. Overall, as a novel biomarker, ctDNA holds substantial promise in advancing clinical practice. This review focuses on the biological characteristics, pre-analytical variables, and downstream analysis methods of ctDNA and summarizes its role across various clinical scenarios in CRC.

## Introduction

According to the GLOBOCAN 2022 report, colorectal cancer (CRC) ranks third in global incidence and second in mortality [1]. And the SEER database reports the 5-year relative survival by stage at diagnosis are 91.1%, 73.7% and 15.7% for localized, regional (spread to regional lymph nodes) and metastatic CRC (mCRC), respectively [2]. A major challenge in the treatment of CRC is that solitary tissue biopsy analysis cannot capture real-time and comprehensive genetic expression features of the tumor. This limitation can be due to (1) heterogeneity of tumor; (2) detected mutations may be results of therapeutic pressure and (3) invasive and expensive procedure of biopsy [3, 4]. Recent advances suggest that liquid biopsy may be a solution to this predicament. This technique involves detecting and analyzing tumor cells, such as circulating tumor cells (CTCs, which are rare in circulation of CRC patients), or their released products, such as extracellular vesicles (EVs), proteins, and tumor-derived circulating cell-free DNA (cfDNA), known as circulating tumor DNA (ctDNA) [5–7]. Among these biomarkers, ctDNA shares the same genetic and epigenetic characteristics as the tumor cells from which it originates, which holds significant potential as a complementary approach to tumor tissue analysis [8]. Its applications include comprehensive molecular profiling of tumor, detection of molecular residual disease (MRD), early cancer detection, treatment response evaluation, and guidance in treatment decisions [9, 10].

In this narrative review, we present an overview of the latest advances in the field of ctDNA analysis. This includes a discussion of the biological characteristics of ctDNA, key considerations for pre-analytical variables, analytical methods, early cancer detection platforms, and the application of ctDNA analysis across various disease contexts.

## Biology of cfDNA

### Origin of cfDNA

As an important component of cfDNA, ctDNA constitutes less than 1% of the total cfDNA [11]. Studies suggest that apoptosis of tumor cells is a primary mechanism for ctDNA release; however, in cases of extensive tumor necrosis, necrosis may become the predominant release mechanism [4, 12–14]. Additionally, studies indicate that, within the tumor microenvironment, cfDNA molecules can also be released into circulation by granulocytes, lymphocytes, germline cells, and even microbiota [4, 15–17]. Recently, Mattox and colleagues reported that in CRC patients, the elevated cfDNA may mainly derive from leukocytes, particularly neutrophils, possibly as a result of a systemic effect induced by the tumor [18]. Active secretion via tumor exosomes has also been proposed as another main mechanism for ctDNA release [19]. Nonetheless, our understanding of the spatial distribution of ctDNA molecules within tumor-derived EVs remains limited [19, 20].

### Factors influencing ctDNA levels in CRC patients

The concentration of cfDNA in the blood of CRC patients varies significantly, ranging from 22 to 3922 ng/ml, while in healthy individuals, it is only 5 to 16 ng/ml [11, 21, 22]. In circulation, free cfDNA molecules are less stable than those bound to proteins, EVs, or nucleosomes [23]. Additionally, studies have reported that the following variables also influence the abundance of cfDNA.

#### Deoxyribonuclease (DNase) activity

In patients with gastrointestinal cancers, Tamkovich and colleagues demonstrated that the activity of DNase is lower than in normal individuals, resulting in a higher concentration of cfDNA in these patients [24]. Additionally, recent studies have shown that tumor burden, tumor cell turnover, the vascularity and spatial location of the tumor can affect DNase activity, thereby influencing cfDNA concentrations [4, 22, 25].

#### Tumor stage, subtype and metastasis site

Detection rates for ctDNA vary across studies, ranging from approximately 40% to 90% for stage I to III CRC, and nearly 100% in patients with mCRC [26–28]. Pathological subtypes also influence ctDNA abundance. CRC patients with mucinous features exhibit lower ctDNA levels than those with adenocarcinoma features, possibly due to mucin acting as a physical barrier that restricts ctDNA release into circulation [25, 29]. In mCRC, ctDNA levels vary depending on the site of metastasis. Patients with liver-only or lymph node-only metastases show higher ctDNA levels than those with lung-only or peritoneum-only metastases [30]. The peritoneum’s poorly vascularized mesothelial tissue may limit ctDNA release, leading to lower detectable levels in cases of peritoneal metastases (PMs) [29, 31]. However, Van’t Erve and colleagues demonstrated that ctDNA levels were higher in peritoneal washing fluid than in plasma for mCRC patients with PMs, indicating that ctDNA detection in peritoneal fluid might provide an alternative method for guiding treatment in these patients [31]. Furthermore, ctDNA levels can be low in patients with early-stage local recurrence, likely due to a relatively low tumor burden [30, 32, 33].

#### Other variables

Research has shown that specific cellular events, such as macrophage phagocytosis and the formation of neutrophil extracellular traps, can influence the ctDNA abundance [16, 34]. In a recent study involving CRC cell lines, Pessei and colleagues observed that tumors with low methylation levels released a higher number of cfDNA molecules compared to those with high methylation levels [35]. Additionally, factors such as demographic characteristics, non-cancerous diseases, individual behaviors, physical conditions, environment exposures and medication use have also been implicated in affecting ctDNA abundance [14, 20].

### Short half-life of ctDNA assists dynamic assessment of treatment response

In circulation, ctDNA molecules are degraded by macrophages, and the degradation products can either be excreted in urine or absorbed by the liver and spleen [24, 36]. The half-life of ctDNA ranges from 16 min to 2.5 h [37]. Therefore, ctDNA may serve as a real-time marker of treatment response, complementing conventional radiological assessments [32, 37].

## Considerations about pre-analytical variables before downstream analysis

A typical cfDNA analysis workflow includes blood collection, plasma-blood cell separation, cfDNA extraction, and downstream analysis [38]. Several pre-analytical variables before downstream analysis can affect the ctDNA yield.

### Matrix and blood collection tube (BCT)

It is widely agreed that plasma, rather than serum, is the optimal matrix for cfDNA extraction [39–41]. The reasons why plasma is preferred over serum are: (1) cfDNA from leukocyte lysis in serum may cause sample contamination; (2) ctDNA in serum samples may partially adhere to the blood clot and (3) the platelet component in blood samples may be lost, while platelets are an important source of tumor-derived nucleic acids [42]. The common amount for blood collection is 15–20 ml [40]. However, some studies suggest that detecting MRD requires a larger volume of blood due to the lower tumor burden. While in patients with relatively high tumor burden, such as during mCRC anti-EGFR rechallenge treatment, a smaller volume of blood may be sufficient for detection [43–45]. The choice of BCTs primarily depends on the time from blood collection to sample processing, as well as the type of downstream analysis [46]. EDTA BCTs are the most commonly used for ctDNA analysis due to their ex vivo ability of DNase inhibition and anticoagulation. Additionally, EDTA BCTs are compatible with subsequent PCR-based ctDNA downstream analysis [47, 48]. When delayed processing (> 6 h) of blood samples is unavoidable, cell-stabilization BCTs are recommended. These BCTs provide both anticoagulation and leukocyte stabilization properties, which are crucial for preventing contamination of ctDNA in the sample. Additionally, these BCTs also allow for long-term storage (> 7 days) at various temperatures (4 °C or room temperature, RT) [46]. Table 1 provides a brief comparison of the parameters for commonly used BCTs developed by BD (USA), Streck (USA), Qiagen (Germany) and PAXgene (Germany) and Roche (USA) for ctDNA analysis [49, 50].

**Table 1:** Comparison of commonly used BCTs for blood collection in ctDNA analysis

Manufacturer	Anti-coagulation	Cell stabilization	Storage length without contamination	Storage temperature	Collection volume (ml)
BD	√	–	~ 6 h	RT	10
Streck	√	√	7 days	RT	10
Qiagen	√	√	7 days	RT	10
PAXgene	√	√	7 days	RT	10
Roche	–	√	14 days	RT	8

*RT* room temperature

### Plasma storage and centrifugation

To maintain the integrity of ctDNA molecules during storage, multiple freeze–thaw cycles, shaking of specimens during transportation, and the presence of air bubbles in BCTs are not recommended. Additionally, it is recommended to aliquot samples throughout the entire analysis process [50, 51]. For short-term storage (typically < 7 days after blood collection), temperature fluctuations from 4 °C to RT don’t affect the integrity of ctDNA [49]. For long-term storage, it is recommended to preserve plasma samples at − 80 °C before subsequent procedure [20]. Sorber and colleagues compared five different plasma centrifugation methods and demonstrated that, regardless of whether EDTA BCTs or cell-stabilization BCTs were used, double centrifugation protocols were associated with high cfDNA concentrations. Double centrifugation protocols are therefore recommended to increase ctDNA yield during plasma extraction [51]. Usually, in the first round of double centrifugation, a speed of approximately 800–1600 g is performed to prevent leukocyte-derived DNA from lymphocytic lysis that reducing signal-to-noise ratio. While in the second round, higher speed of approximately 14,000–16,000 g will be performed to remove cellular debris and other contaminants [51–53].

### cfDNA extraction

Silica membrane columns and magnetic beads-based technologies share similar extraction quantity, quality, and availability regardless of manual or automatic protocols [46]. Commercial cfDNA extraction kits are also developed for various downstream analysis in order to obtain higher yield of ctDNA or recovery rates of short-length DNA fragments, and the use of commercial kits is more convenient and reliable in clinical routine practice [20, 46].

Pre-analytical variables before downstream analysis are affected by the complex nature of ctDNA, which imposes specific requirements on sample handling. Despite the challenges encountered, ensuring sufficient yield and integrity of ctDNA, as well as establishing standardized sample processing protocols to achieve comparable results in multicenter studies, is crucial for downstream analysis. Expert panels are continuously working to establish standardized protocols for these pre-analytical variables [20, 50].

## PCR- and NGS-based techniques: common methods for ctDNA downstream analysis

Downstream analysis of ctDNA requires highly sensitive detection methods [44, 54]. PCR- and NGS-based methods are widely used for downstream analysis of ctDNA, with PCR methods having a limit of detection (LoD) of 0.01%–0.1% [55]. The LoD for NGS methods varies depending on the specific methodology, with some methods achieving sensitivities as low as 0.01% [5, 39]. Across different studies, commonly detected molecular abnormalities include single nucleotide variations (SNVs), insertions/deletions (indels), copy number variations (CNVs), and chromosome rearrangements (CRs) [55, 56]. Various new detection methods are currently being developed and validated, Table 2 lists some of them and provides a brief comparison [22, 25, 28, 57–70].

**Table 2:** Comparison of commonly used ctDNA downstream analysis for detection of molecular alterations

Methods	LoD	Advantages	Disadvantages	Variation type
PCR-based detection				
qPCR [63]	0.1%	Rapid turnaround time Relatively lower cost High analytical sensitivity	Limited to known-mutation detection Restricted multiplex detection capability Dependence on specific probes	SNV, indel
ddPCR [64] BEAMing [22]	0.001% 0.01%			
NGS-based detection				
Targeted sequencing				
CAPP-Seq [65]	0.01%	Multiplex detection capability Novel mutation identification High analytical sensitivity	Complex preanalytical procedure and bioinformatic analysis Higher cost Longer turnaround time	SNV, indel, CNV, CR
Safe-Seq [66]	0.1%			SNV, indel
TEC-seq [25]	0.1%			SNV, indel
Guardant360 [67]	< 0.1%			SNV, indel, CNV, CR
FoundationOne [71] *	> 0.5% ≥20%			SNV, indel, CNV, CR, MSI
Non-targeted sequencing				
WES [69]	1%	Unbiased genetic alteration detection Comprehensive structural variant analysis High-throughput genomic profiling	Lower sensitivity Complex bioinformatic analysis Longer turnaround time Very high cost	SNV, indel, CNV, CR
WGS [70]	5%			SNV, indel, CNV, CR

*qPCR* quantitative PCR; *ddPCR* digital droplet PCR; *BEAMing* Beads, Emulsion, Amplification, Magnetics; *CAPP-Seq* cancer personalized profiling by deep sequencing; *Safe-Seq* safe sequencing system; *TEC-seq* targeted error correcting sequencing; *WES* whole-exome sequencing; *WGS* whole-genome sequencing; *LoD* Limit of Detection; *SNVs* single nucleotide variations; *Indels* insertion/deletions; *CNV* copy number variation; *MSI* microsatellite instability; *CR* chromosome rearrangement

^*^ For FoundationOne, sensitivity is > 0.5% for detecting SNVs, indels, and CRs, while it is ≥ 20% for detecting CNVs

### PCR-based methods

Quantitative PCR (qPCR) is a fast, cost-effective, and straightforward method for the relative quantification of somatic mutations, with an LoD of ≤ 10% [72]. Advanced qPCR techniques, such as COLD-PCR, can achieve an LoD of 0.1% for detecting *KRAS* mutations in CRC [73]. Digital droplet PCR (ddPCR) is a more sensitive, rapid, simple, and cost-effective method for the absolute quantification of extremely rare tumor somatic mutations in ctDNA, with an LoD of 0.1% [40, 74]. BEAMing (Beads, Emulsion, Amplification, Magnetics), which combines ddPCR, magnetic beads, and flow cytometry, has demonstrated high performance in detecting extremely low-frequency mutations in ctDNA, achieving an LoD of 0.01% [22, 34]. However, to ensure optimal assay efficiency and minimize sampling bias, the DNA input for analysis typically needs to exceed 60ng to achieve an LoD of 0.01%, which may be a potential pitfall of this method [75]. Additionally, the specificity and sensitivity of ddPCR are closely related to the design and characteristics of the probes used in the analysis [76].

### NGS-based methods

Within a single run, NGS-based methods can sequence millions of DNA fragments and genes of interest. However, unlike tissue specimen sequencing, detecting low-frequency mutations in plasma using NGS requires deeper sequencing depth and the use of techniques such as molecular barcodes to reduce sequencing errors [77]. They can be divided into: (1) targeted detection methods, which detect pre-specified gene panels; while (2) non-targeted methods, which detect the entire genome without prior knowledge of tumor molecular information to discover new molecular changes in tumors [37].

#### Targeted methods

Cancer personalized profiling by deep sequencing (CAPP-Seq) is an ultra-sensitive technology that utilizes hybrid capture, highly selective probes, and enhanced bioinformatic algorithms to improve analytic sensitivity [65, 78]. Safe sequencing system (Safe-Seq) combines unique molecular barcode and redundant sequencing to increase analytic sensitivity and reduce sequencing errors. Therefore, it can detect single mutated molecule within approximately 5 × 10^3^–1 × 10^9^ wild-type molecules, that has ability to detect extremely-low-frequency mutations with high sensitivity [66, 79]. Guardant360 employs hybridization capture and digital sequencing to enhance sensitivity, incorporating bioinformatics and molecular barcodes to minimize sequencing errors. As a result, it achieves an LoD of 0.1% and demonstrates high accuracy across multiple prospective studies [67, 71, 80–84]. FoundationOne also uses advanced bioinformatic algorithms to improve accuracy and can detect alterations such as base substitutions, indels, CNVs, chromosome rearrangements, microsatellite instability (MSI), and blood-derived tumor mutational burden (bTMB) [71].

#### Non-targeted methods

Whole-genome sequencing (WGS) and whole-exome sequencing (WES) can both detect pathogenic mutations without prior knowledge of molecular abnormalities of the tumor. Compared to WES, WGS provides more comprehensive genome coverage [69, 70, 85]. Although these methods are associated with higher costs and relatively lower sensitivity, they are more suitable for detecting CNVs and CRs compared to targeted sequencing methods [86]. These non-targeted sequencing technologies have been applied to MRD detection and may play a key role in identifying tumor with unique driver mutations. Additionally, they can provide critical support for selecting candidate genes for customized small-panel deep sequencing MRD strategies [87].

Various ctDNA analysis methods are now available for CRC patients, offering real-time tumor genetic insights to guide clinical practice. However, for ctDNA analysis to become widely applicable in clinical settings, establishing a standardized workflow is essential. This requires large-scale, multi-center comparative studies to confirm its feasibility. Additionally, rigorous quality control throughout the entire analytical process is crucial to ensure oncologists receive accurate data, enabling well-informed clinical decisions [50].

## Multi-analyte- and epigenetics-based ctDNA analysis platforms can detect early-stage cancers

The HUNT study demonstrated that CRC-related methylated ctDNA markers can be detected up to 2 years before clinical diagnosis, supporting the hypothesis that ctDNA detection can identify early-stage cancers [88]. ctDNA can be detected in ≥ 70% of localized CRC cases, and most pathogenic alterations reflecting tumorigenesis and tumor progression can also be identified in ctDNA, highlighting the potential value of ctDNA analysis in cancer screening [25, 27, 89, 90]. The Guardant Shield test was recently approved by the FDA as the first blood-based screening tool for CRC in average-risk adults aged 45 and older. This cfDNA test demonstrates a sensitivity of 83.1% (95% CI 72.2%–90.3%) for detecting stage I to III CRC and a sensitivity of 13.2% (95% CI 11.3%–15.3%) for identifying advanced precancerous lesions [91]. In Table 3, we briefly introduce selected multi-analyte and epigenetics-based platforms for this purpose. However, as suggested by the early termination of the COBRA trial (NCT01172639), genetic mutation analysis alone is insufficient to reduce false positives. The integration of additional techniques, such as fragmentomics and mutational signature analysis, is necessary to enhance the sensitivity, specificity, and LoD of the assays [92–96]. Moreover, there are other issues that need to be addressed before ctDNA testing can be applied to early cancer screening: (1) The sensitivity for detecting precancerous lesions remains low. A potential solution could be the combination of ctDNA with other types of tumor markers for joint detection; (2) Precancerous colorectal lesions also exhibit significant heterogeneity and typically do not release their DNA molecules into circulation, so ctDNA analysis may not accurately distinguish such patients from healthy individuals; (3) False positives may increase the cost of unnecessary subsequent examinations. For example, aging and solitary methylated *BCAT1* in ctDNA may lead to false-positive results in CRC diagnosis [97, 98].

**Table 3:** Comparison of ctDNA analysis platforms for early cancer screening based on different principles

Platform	Detected biomarker	Analysis method	Performance
Methylation-based platforms			
EpiproColon [99]	methylated *SPET9*	PCR	Detect CRC at early-stage
Cd-score [100]	Nine methylation biomarkers identified through machine learning	Deep sequencing	CRC screening Prognostic prediction
ColonES [10]	191 methylation haplotype biomarkers identified through machine learning	MethylationNGS	Detect CRC at early-stage Prognosis prediction
Multi-analyte-based platforms			
CancerSEEK [101]	ctDNA and protein biomarkers	Multiplex-PCR	Discriminate eight common malignancies at early-stage and determine their tissue of origin
LIFE-CNA [102]	ctDNA fragmentation, CNV, and chromatin signature identified through machine learning	WGS	Detect CRC at early-stage; Response evaluation
LUNAR-2 [103]	ctDNA somatic mutation, methylation, and fragmentomic pattern	NGS	Detect CRC across stages; Average risk population screening

## The roles of ctDNA in post-operative contexts of CRC

The MOSAIC study has demonstrated that risk stratification based solely on clinicopathologic factors for post-resection treatment does not improve survival [104]. The commonly accepted explanation for this finding is the presence of MRD, which is considered a critical cause of relapse [104–106]. Growing evidence suggests that ctDNA analysis can detect MRD and predict disease relapse following definitive treatment for solid tumors, offering significant potential for guiding personalized adjuvant therapies [107].

### Strategies of MRD detection

The detection of MRD through ctDNA analysis can be categorized into two approaches: tumor-informed and tumor-agnostic methods [108].

#### Tumor-informed approaches

Currently, the common tumor-informed analysis pipeline involves obtaining tumor genomic features by performing WES or large-panel NGS on biopsy or surgical specimens. Combined with primary tumor driver genes, a tumor-specific panel is established, which can be used for highly sensitive MRD detection through ultra-deep PCR or NGS [108, 109]. This approach leverages prior knowledge of the tumor genomic features to reduce the number of genes in the tumor-specific panel, followed by ultra-deep sequencing of plasma samples (such as variant allele frequency (VAF) < 0.01%), which is ideal for detecting very low levels of MRD.

As an example, Reinert and colleagues explored the association between ctDNA positivity and recurrence in stage II CRC using Signatera [26]. Following tumor tissue NGS with a panel which consists of 1042 SNV sites, panels with 16 high-ranked tumor-specific SNVs and short indels were designed for each patient. MRD detection was performed by a NGS sequencing platform (HiSeq 2500 system), that sequencing depth and LoD reaches > 105, 000X per amplicon and 0.001% VAF, respectively. ctDNA positivity was defined as at least two variants are detected within one plasma sample [26].

However, these methods still have limitations: (1) Solitary biopsy cannot fully capture the genomic landscape of the tumor; (2) The genomic features of tumor may change over time due to disease progression and therapeutic pressure, potentially leading to false-positive results; (3) The selection of tumor variants and sequencing depth for tumor-specific panels are subjective. These decisions are often made by bioinformatic algorithms, which may overlook driver mutations while detecting passenger mutations. The determination of ctDNA positivity is also subjective; for example, aforementioned Signatera sets a threshold of ≥ 2 variants, whereas Safe-Seq uses a threshold of ≥ 1 variant; (4) Biopsy is an invasive procedure and costly [32, 108, 109].

#### Tumor-agnostic approaches

These methods do not require prior knowledge of the tumor genomic landscape but use a fixed gene panel to detect mutations in the target region, which limits the sequencing depth. Compared to tumor-informed approaches, this method has lower sensitivity and a higher false-positive rate [108, 109].

Some strategies can optimize the accuracy of tumor-agnostic ctDNA detection: (1) Using larger gene panels and optimized bioinformatic algorithms. For example, Chen and colleagues established a 425-gene panel (Geneseeq Prime) for quantifying mutation abundance in ctDNA, with a sequencing depth of 4693X. ctDNA positivity was defined when the number of true variants exceeded 5% of all tracked variants [106]. (2) Integrating multiple sequencing techniques, such as Guardant Reveal, which integrates the detection of ctDNA mutations and ctDNA methylation levels, achieving an LoD as low as 0.01%. In a prospective study by Parikh and colleagues, Guardant Reveal demonstrated a sensitivity of 91% and a specificity of 100% for MRD detection [110]. (3) Using more sensitive PCR techniques [108]. For example, the OncoBEAM RAS CRC kit uses highly sensitive BEAMing technology for detecting *RAS* mutations in the plasma of mCRC patients [111]. Overall, these tumor-agnostic methods offer advantages such as reducing turnaround time, lowering costs, and enabling large-scale detection of suspicious mutations [109].

In clinical research, the choice between tumor-informed and tumor-agnostic strategies primarily depends on the availability of tumor tissue, cost-effectiveness, and the robustness of the specific MRD detection methods used [8, 109].

### The roles of MRD in guiding adjuvant therapy, monitoring response and prognostication

Whether adjuvant chemotherapy (ACT) is necessary for CRC after surgery remains controversial. Better predictive biomarkers are needed to help select patients who can benefit from ACT [112].

The DYNAMIC study is the first to use ctDNA analysis for risk stratification in determining the necessity for ACT in stage II colon cancer patients. In ctDNA-guided group, there was nearly a 50% reduction in the use of ACT compared to standard-management group (15% vs. 28%, RR = 1.82, 95% CI 1.65–2.65). Importantly, the reduce of ACT administration did not compromise 2-year and 3-year relapse-free survival (RFS) rates (2-year RFS, 93.5% vs. 92.4%; 3-year RFS, 91.7% vs. 92.4%, in ctDNA-guided group and standard-management group, respectively), suggesting non-inferiority of the ctDNA-guided approach in this setting [113]. The AGITG DYNAMIC-Rectal study also evaluated the role of ctDNA in guiding ACT for patients with locally-advanced rectal cancer (LARC). The results showed that significantly fewer patients in the ctDNA-guided group received ACT compared to the standard management group (46% vs. 77%, P < 0.001), leading to comparable 3-year RFS rates between the ctDNA-guided and standard-management group [114].

Emerging studies indicate that changes in ctDNA after treatment can serve as markers for therapeutic efficacy and prognosis during ACT. The GALAXY study categorized ctDNA changes at postoperative weeks 4 and 12 during ACT into four patterns: persistently negative, positive to negative, negative to positive, and persistently positive. The 18-month disease-free survival (DFS) rates for these four patterns were 92.1%, 81.4%, 33.8%, and 22.9%, respectively. This indicated that persistent ctDNA positivity or a shift from negative to positive during ACT was a strong predictor of recurrence [115]. The study also introduced “ctDNA clearance”, defined as the time interval between the post-surgery ctDNA-positive result and the first ctDNA-negative result, regardless of subsequent ctDNA status. Findings revealed that ACT was associated with a higher cumulative incidence of ctDNA clearance (adjusted HR = 8.50, 95% CI 4.2–17.3; P < 0.0001) and prolonged DFS (adjusted HR = 11.0, 95% CI 5.2–23.0, P < 0.0001), suggesting potential benefits of ACT [115]. In another study on stage III CRC, Henriksen and colleagues classified exponential changes in ctDNA levels during ACT into two patterns: a fast-growth pattern with a 143% monthly increase, and a slow-growth pattern with a 25% monthly increase. The results revealed that the fast-growth pattern was associated with recurrence (HR = 2.7; 95% CI 1.1–6.7; P = 0.039) and shorter overall survival (OS) (HR = 42.0; 95% CI 8–221; P < 0.001) [116].

In addition, ongoing clinical studies, including IMPROVE (NCT03637686), IMPROVE-IT2 (NCT04084249), PEGASUS (NCT04259944) and NRG-GI005 (NCT04068103) are investigating the role of ctDNA in guiding initial selection of adjuvant treatment regimens and making adjustments during therapy.

Regarding neoadjuvant therapy (NAT) for LARC, the total neoadjuvant therapy strategy has become the current primary recommended approach, making precise baseline risk stratification to guide individualized NAT a key research focus [117]. Our team is conducting a randomized controlled trial (CINTS-R, NCT05601505) to assess the value of ctDNA in guiding NAT decisions for LARC patients. Regarding the organ-preservation strategy, current evidence suggests that ctDNA alone, due to sensitivity limitations, cannot fully assess tumor residue for the “watch and wait” strategy. Future research should focus on improving ctDNA sensitivity and specificity and integrating multi-omics, clinical pathology, and imaging to better predict complete tumor response, optimizing the watch-and-wait approach [118].

## The roles of ctDNA analysis in metastatic disease

At baseline, ctDNA analysis helps with risk stratification for patients with mCRC [119]. For instance, the PLACOL study indicated that baseline ctDNA levels below 0.1ng/ml were associated with better treatment response and longer OS for mCRC patients receiving a first- or second-line chemotherapy (6.8 vs. 33.4 months; adjusted HR = 5.64; 95% CI 2.5–12.6; P < 0.0001) [120]. Parikh and colleagues proposed that early changes in ctDNA levels during treatment can reflect therapeutic efficacy earlier than conventional imaging methods, thereby aiding in timely adjustments of treatment strategies [121]. Moreover, ctDNA provides more comprehensive and detailed molecular information than tissue biopsies [119]. For example, in the context of anti-*EGFR* therapy, the TTD-ULTRA and CAPRI-GOIM studies both validated the feasibility of determining *RAS* status through ctDNA analysis [122–125]. The CRICKET and CHRONOS studies also showed that determining *RAS* gene status through ctDNA to select appropriate patients for anti-*EGFR* rechallenge therapy is feasible [126, 127]. Additionally, the VELO study demonstrates that wild-type *RAS/BRAF* ctDNA prior to rechallenge is associated with a better disease control rate [128]. Furthermore, ctDNA can reveal acquired mutations that drive resistance to targeted therapies [119]. The PARADIGM study suggested that gene alterations associated with resistance to panitumumab could be detected in baseline ctDNA, including *KRAS*, *NRAS*, *PTEN* and *EGFR* extracellular domain mutations, *HER2* and *MET* amplifications, and *ALK*, *RET* and *NTRK1* fusions [129, 130]. The EVICT study further investigated mechanisms of drug resistance in *BRAF V600E*-mutated mCRC, identifying polyclonal mutations in *KRAS*, *NRAS*, and *MAP2K1*, along with *MET* amplification [131]. In the TRIUMPH study on *HER2*-amplified mCRC, gene alterations linked to drug resistance were also identified [84].

## The roles of ctDNA analysis in immunotherapy of CRC

Immunotherapy has been accepted for one of the first choices of dMMR/MSI-H and/or TMB-H CRC. Several studies have explored the value of ctDNA in immunotherapy. In a proof-of-concept study, Cabel and colleagues evaluated whether ctDNA could monitor treatment response in patients, including those with MSI CRC, treated with nivolumab or pembrolizumab. At week 8, changes in ctDNA levels were significantly correlated with changes in tumor size (R = 0.86; P = 0.002). Patients with undetectable ctDNA at week 8 exhibited marked and lasting treatment responses, and ctDNA status was an important predictor of PFS and OS [132]. The INSPIRE study conducted serial ctDNA assessments in 94 patients with advanced solid tumors receiving pembrolizumab. The results showed that an early increase in ctDNA levels could predict non-response to immunotherapy with 100% specificity. Patients who achieved ctDNA clearance during treatment had a 100% OS rate, with a median follow-up exceeding 25 months, whereas an increases in ctDNA level indicated a lack of objective response [133].

In addition, the application value of bTMB and ctDNA-based MSI (bMSI) has been gaining increasing attention. The CO.26 trial demonstrated that bTMB, particularly when derived from clonal and subclonal mutations, may serve as a useful biomarker for identifying CRC patients likely to respond to immunotherapy, whereas traditional tissue-based TMB did not show such efficacy [134]. In the ARETHUSA trial, tissue biopsies and ctDNA analysis of *MGMT*-methylated mCRC patients pretreated with temozolomide (TMZ) revealed distinct mutational signatures and increased bTMB following TMZ treatment. Furthermore, some patients with specific mutations achieved disease stabilization after receiving pembrolizumab [135]. Additional studies have shown that bMSI testing can reliably identify MSI-H patients who can benefit from immunotherapy [136, 137].

Currently, ctDNA analysis can reflect tumor burden, predict treatment response, and monitor tumor evolution during treatment. However, the main drawback of ctDNA analysis in clinical practice is the issue of “low shedders”, which we have discussed above. In such cases, ctDNA is unlikely to serve as an effective biomarker, which may lead to false-negative results in risk stratification and increase the risk of incorrect treatment decisions. Therefore, in current clinical practice, not all patients with negative ctDNA results can be classified as “low-risk”. Future improvements can focus on enhancing the sensitivity of ctDNA analysis methods, combining detection in serous cavity effusions, and establishing optimized stratification strategies [112, 138].

## Conclusions and perspectives

As a biomarker of significant value in liquid biopsy, ctDNA has demonstrated considerable potential in the management of CRC. Its main advantages include providing a non-invasive, real-time, dynamic, and comprehensive method for assessing tumor burden, effectively monitoring cancer progression and treatment response. Furthermore, ctDNA, as a key tool for precision medicine, offers valuable molecular genetic information that supports the development of precise treatment strategies. Studies have shown that ctDNA plays an important role in detecting MRD, monitoring disease recurrence, predicting treatment efficacy and prognosis, and guiding individualized therapies in CRC. However, ctDNA analysis faces challenges due to its complex biological nature and technical limitations, particularly regarding sample handling, detection methods, and data interpretation. These issues require further refinement and standardization, and validation through large-scale clinical studies. With ongoing technological advancements and further research, ctDNA is expected to play an increasingly important role in more clinical scenarios in CRC management.

## Data Availability

No datasets were generated or analysed during the current study.
